# A Novel Blood-Based microRNA Diagnostic Model with High Accuracy for Multi-Cancer Early Detection

**DOI:** 10.3390/cancers14061450

**Published:** 2022-03-11

**Authors:** Andrew Zhang, Hai Hu

**Affiliations:** 1 Del Norte High School, San Diego, CA 92127, USA; andrewzhang20052005@gmail.com; 2 Chan Soon-Shiong Institute of Molecular Medicine at Windber, Windber, PA 15963, USA; 3 miRoncol Diagnostics, San Diego, CA 92127, USA

**Keywords:** multi-cancer early detection, microRNA, noninvasive, blood-based diagnostic model

## Abstract

**Simple Summary:**

Development of noninvasive diagnostic tests capable of detecting multiple cancer types early is urgently needed. Majority of the tests that are currently in development for multi-cancer early detection are based on next generation sequencing technology to evaluate methylation or fragmentation patterns of circulating tumor DNAs. Here, we developed a serum-based 4-microRNA diagnostic model, which when compared to the existing next generation sequencing-based tests, demonstrated superior performance in detecting 12 cancer types in the largest case-control validation cohort to date. The simplicity of our model also makes it feasible to develop an in vitro diagnostic (IVD) test capable of decentralized testing, supporting the wide adoption and compliance in the at-risk general population.

**Abstract:**

Early detection is critical to reduce cancer deaths as treating early stage cancers is more likely to be successful. However, patients with early stage diseases are often asymptomatic and thus less likely to be diagnosed. Here, we utilized four microarray datasets with a standardized platform to investigate comprehensive microRNA expression profiles from 7536 serum samples. A 4-miRNA diagnostic model was developed from the lung cancer training set (*n* = 416, 208 lung cancer patients and 208 non-cancer participants). The model showed 99% sensitivity and specificity in the lung cancer validation set (*n* = 3328, 1358 cancer patients and 1970 non-cancer participants); and the sensitivity remained to be >99% for patients with stage 1 disease. When applied to the additional combined dataset of 3792 participants including 2038 cancer patients across 12 different cancer types and 1754 independent non-cancer controls, the model demonstrated high sensitivities ranging from 83.2 to 100% for biliary tract, bladder, colorectal, esophageal, gastric, glioma, liver, pancreatic, and prostate cancers, and showed reasonable sensitivities of 68.2 and 72.0% for ovarian cancer and sarcoma, respectively, while maintaining 99.3% specificity. Our study provided a proof-of-concept data in demonstrating that the 4-miRNA model has the potential to be developed into a simple, inexpensive and noninvasive blood test for early detection of multiple cancers with high accuracy.

## 1. Introduction

The World Health Organization (WHO) estimated that there were 19.3 million new cancer cases and 10 million cancer deaths worldwide in 2020, with most new cases from breast (11.7%), lung (11.4%), colorectal (10.0%), prostate (7.3%), and stomach (5.6%) cancers [1]. Cancer survival rates decrease significantly if cancer is diagnosed at late stages. For example, the five-year survival rate for localized lung cancers is 59%, but is only 6% for metastatic diseases [2]. Unfortunately for many cancers, patients with early stage diseases are typically asymptomatic and thus less likely to be diagnosed [3,4]. Therefore, detection of cancer at early stages is critical to decrease cancer-related mortality as treatment is more likely to be successful at early stages.

Currently, the United States Preventive Service Task Force (USPSTF) only recommends four cancer screenings in the general population: breast cancer mammography for women aged 50–74, cervical cancer cytology/HPV testing for women aged 21–65, colon cancer colonoscopy and/or stool based testing for persons aged 50–75, and lung cancer low-dose CT scans in smokers aged 50–80 [5,6,7,8]. However, adoption and adherence to these screening guidelines varies widely [9,10]. The other commonly used screening method is the testing of prostate-specific antigen (PSA) for prostate cancer. However, PSA screening suffers from false positive results resulting in invasive prostate biopsy, overdiagnosis, and overtreatment, and thus is not uniformly recommended by USPSTF [11]. Due to the lack of effective screening methods for most cancer types, two-thirds of all cancer diagnoses and three-fifths of all cancer deaths are not covered by the existing evidence-based cancer screening guidelines that address a single cancer at a time [2]. In addition, if these guidelines are followed sequentially, the cumulative risk of false positives could increase substantially to greater than 50% [12]. Thus, there is an urgent unmet need to develop a test capable of detecting multiple cancer types early and simultaneously, ideally noninvasive, such as a blood test, which has become the cornerstone of the so-called multi-cancer early detection (MCED) paradigm. Such a MCED test often requires very high specificity, preferably >99%, to ensure minimum false positives in order to be able to screen the at-risk general population.

Molecules such as microRNAs (miRNAs) may serve as biomarkers for MCED. miRNAs are small single-stranded non-coding RNA molecules of an average of 22 nucleotides long. miRNAs function in negative post-transcriptional regulation of gene expression primarily by binding with complementary sequences in the 3′ untranslated region (3′ UTR) of mRNA molecules [13]. miRNAs appear to regulate more than 50% human genes, and abnormal expression of miRNAs has been implicated in many human cancers [14]. miRNAs are also abundant as extracellular circulating molecules released into circulation by tumor cells either through cell death or by exosome-mediated signaling [15]. Combined with its remarkable stability in the blood and other body fluids, circulating cell free miRNAs have the potential to serve as noninvasive biomarkers for cancer screening and diagnosis [16].

In the study reported here, we described the development and validation of a circulating miRNA-based diagnostic signature for MCED by utilizing four large miRNA microarray datasets, all based on a standardized microarray platform.

## 2. Materials and Methods

### 2.1. Study Design

Four microarray datasets totaling 7536 unique participants including 3604 cancer patients and 3932 non-cancer controls were included in the current analysis, all derived from studies originating from a Japanese nationwide research project “Development and Diagnostic Technology for Detection of miRNA in Body Fluids” designed to characterize serum miRNAs in over 50,000 participants across 13 cancer types using a standardized microarray platform [17,18,19,20]. The four datasets were originally assembled to develop diagnostic signatures for lung (GSE137140), ovarian (GSE106817), liver (GSE113740), and bladder (GSE113486) cancers, respectively.

The lung cancer dataset has the largest sample size for a single cancer type (*n* = 1566) and non-cancer controls (*n* = 2178). The original lung cancer study established a 2-miRNA diagnostic model (referred to as the “original 2-miRNA model” in this study) with high sensitivity and specificity for the detection of lung cancer [17]. The objective of the current study was initially set to use this dataset to develop and validate a new diagnostic model that may out-perform the original 2-miRNA model for lung cancer detection. As datasets for additional cancer types were identified, the new model was then evaluated for its performance to detect other cancers.

### 2.2. Participants and Serum Samples

Serum sample collection has been previously described in the original publications [17,18,19,20]. Briefly, serum samples were collected from cancer patients who were referred or admitted to the National Cancer Center Hospital (NCCH) between 2008 to 2016 prior to surgical operation, and stored at 4 °C for one week before being stored at −20 °C until further use. Cancer patients who were treated with preoperative chemotherapy and radiotherapy prior to serum collection were excluded. The serum samples for non-cancer controls who had no history of cancer and no hospitalization during the previous 3 months were collected along with routine blood tests from outpatient departments of three sources: NCCH, National Center for Geriatrics and Gerontology (NCGG) Biobank, and Yokohama Minoru Clinic (YMC). Serums collected from NCCH were stored in the same way as the cancer patients, while those from NCGG and YMC were stored at −80 °C till use. The original studies were approved by the NCCH Institutional Review Board, the Ethics and Conflict of Interest Committee of the NCGG, and the Research Ethics Committee of Medical Corporation Shintokai YMC. Written informed consent was obtained from each participant.

### 2.3. miRNA Microarray Expression Analysis

Details about microarray analysis were described in the original publications [17,18,19,20]. Briefly, total RNA was extracted from 300 μL serum, labeled by 3DGene^®^ miRNA Labeling kit and hybridized to 3D-Gene^®^ Human miRNA Oligo Chip (Toray Industries, Kanagawa, Japan) designed to investigate 2588 miRNA sequences registered in miRBase release 21 (http://www.mirbase.org/, accessed on 10 January 2022). The following low-quality samples were excluded: coefficient of variation of negative control probes >0.15; and number of flagged probes identified by 3D-Gene^®^ Scanner as “uneven spot images” >10. The presence of a miRNA was determined when signal intensity was greater than mean plus two times standard deviation of the negative control signals, and in using the negative control signals the top and bottom 5% of the ranked signal intensities were removed. Background subtraction was performed by subtracting the mean signal of negative control signals (after removing top and bottom 5% as ranked by signal intensities) from the miRNA signal. Normalization across microarrays was achieved by calibrating according to three pre-selected internal control miRNAs (miR-149-3p, miR-2861, and miR-4463).

### 2.4. Diagnostic Model Development

**Figure 1:**
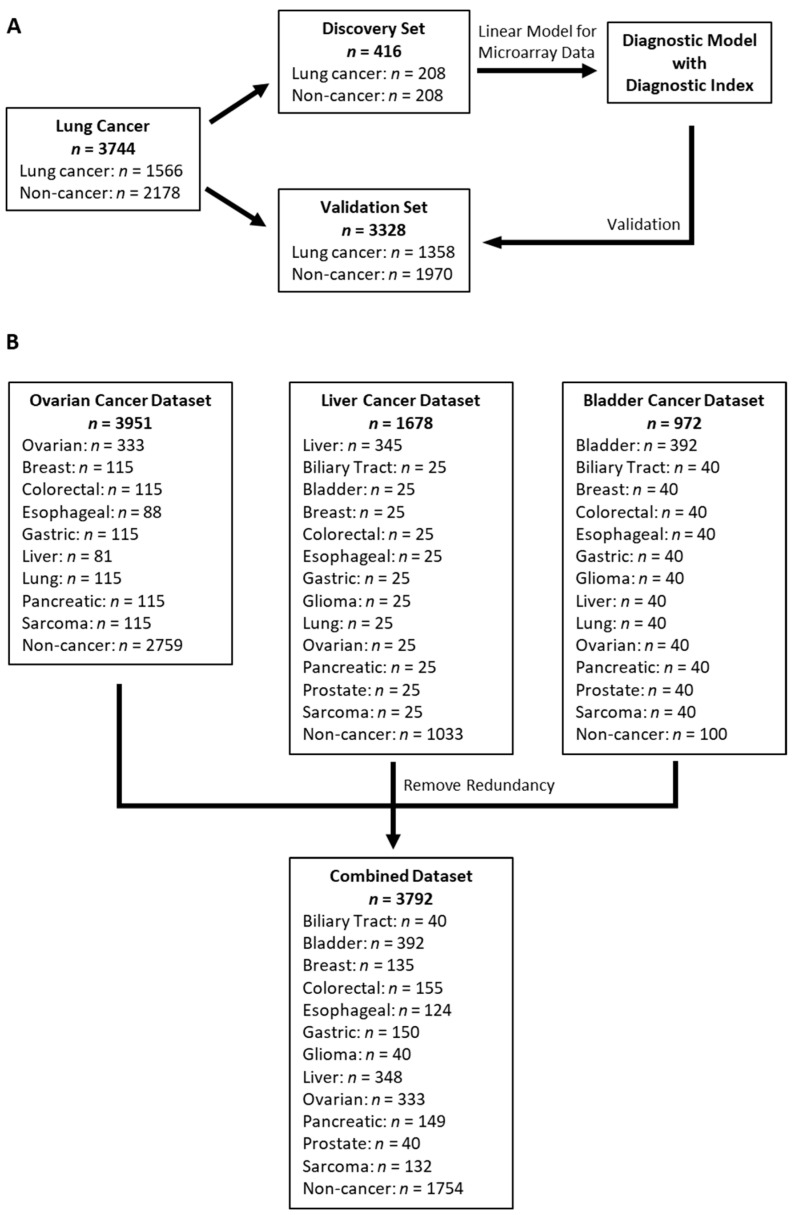
Case flow diagram. (**A**) Lung cancer dataset was split into a discovery and a validation set; (**B**) Ovarian, liver and bladder cancer datasets were combined into a single validation dataset after removing redundant samples.

Patients in the lung cancer dataset were divided into the same discovery and validation sets as in the original publication (Figure 1A) [17], because (1) the discovery set was selected by the original authors to be balanced between cancer and non-cancer with respect to age, sex, and smoking history; (2) 50% of non-cancer participants in the discovery set were from NCCH with the same serum storage condition as cancer patients to minimize potential bias in miRNA candidates selection; (3) Using the same discovery and validation sets allows direct performance comparison of the new diagnostic model with the original 2-miRNA model. As the diagnostic model was developed from the lung cancer discovery set, after its validation in the lung cancer validation set, we further tested its ability as a multi-cancer diagnostic model in a combined dataset of other additional cancer types that were not used in the model development. 

Linear Model for Microarray Data (limma) [21] was performed in the discovery set to evaluate the statistical significance of differential miRNA expression between lung cancer vs. non-cancer. Ten-fold cross validation in the discovery set, based on the area under the curve (AUC) of the Receiver’s Operating Characteristics (ROC) curve analysis, was performed to determine the optimal number of miRNAs for the best diagnostic model. A diagnostic index was calculated as a linear sum of miRNA expression levels weighted by limma statistics. The cut-point for the diagnostic index was chosen to ensure no misclassification of non-cancer controls in the discovery set to minimize false positives as the diagnostic model may potentially be used as a screening test in the at-risk general public.

### 2.5. Statistical Analysis

The diagnostic performance for identifying cancer vs. non-cancer was determined by AUC of the ROC curve analysis, sensitivity, and specificity. Comparing AUC of two ROC curves was done with roc.test function with bootstrapping method from pROC package. Comparing paired sensitivities for the lung cancer clinical subsets was performed by McNemar test. limma analysis was carried out using Bioconductor package limma (http://www.bioconductor.org, accessed on 27 August 2020) [21]. All statistical analysis was performed using R version 4.0.5 (http://www.r-project.org, accessed on 15 July 2020).

## 3. Results

### 3.1. Participants and Datasets

The lung cancer dataset included 1566 lung cancer patients and 2178 non-cancer controls (Figure 1A) [17]. The ovarian cancer dataset consisted of 333 ovarian cancer patients and 2759 non-cancer controls, as well as patients with breast, colorectal, esophageal, gastric, liver, lung, pancreatic, and sarcoma cancers (Figure 1B) [18]. The liver and bladder cancer datasets included 345 liver cancer/1033 non-cancer and 392 bladder cancer/100 non-cancer participants, respectively, in addition to patients with biliary tract, breast, colorectal, esophageal, gastric, glioma, lung, ovarian, pancreatic, prostate, and sarcoma cancers (Figure 1B) [19,20]. With the lung cancer dataset left intact, redundant samples within the other three datasets that showed correlations either among themselves or with samples in the lung cancer dataset being greater than 0.99 were removed. Then, the unique samples from the ovarian, liver, and bladder cancer datasets were then combined into a single non-lung cancer dataset with a total of 3792 samples, including 2038 cancer patients across 12 cancer types and 1754 non-cancer controls (Figure 1B).

The lung cancer dataset was divided into the same discovery set (*n* = 416) and validation set (*n* = 3328) as the original study (Figure 1A). The discovery set included 208 lung cancer patients and 208 non-cancer controls, matched by age, sex, and smoking status [17]. The validation set included 1358 lung cancer patients and 1970 non-cancer controls. The patients with lung cancer included 57% male, 62% former or current smokers, 78% adenocarcinoma, 14% squamous carcinoma, 72% stage I, 15% stage II, and 13% stage III (Table 1).

The 392 bladder cancer patients were of mean age 68 y, 72% male, 5% metastatic, 12% nodal positive, 77% T2 or below, and 80% high grade (Table 1). The 333 ovarian cancer patients were of mean age 57 y, 25% stage I, 10% stage II, 55% serous, 19% clear cell, and 13% endometrioid histology (Table 1). The 348 liver cancer patients were of mean age 68 y, 78% male, 37% stage I, and 33% stage II (Table 1). No detailed demographic information and tumor characteristics for the other cancers were provided by the original studies.

### 3.2. Development of Diagnostic Model

**Figure 2:**
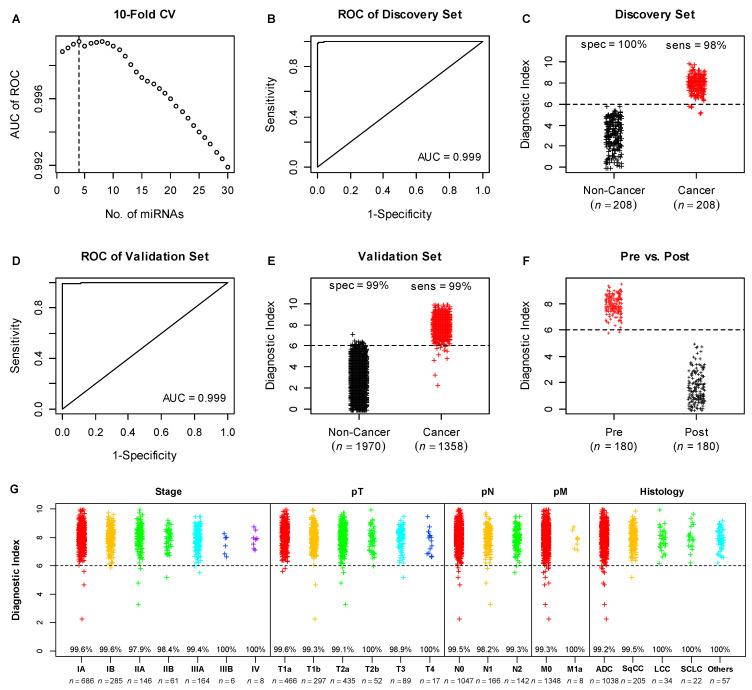
Development and validation of the 4-miRNA diagnostic model in the lung cancer data set. Where applicable, different colors were used to denote different subject conditions. Dotted horizontal lines represent the cut-point for the diagnostic index of our model. (**A**) determination of the optimal number (dotted line) of miRNAs for the diagnostic model by 10-fold cross validation in the discovery set; (**B**) ROC analysis in the discovery set; (**C**) distribution of diagnostic index in the discovery set; (**D**) ROC analysis in the validation set; (**E**) distribution of diagnostic index in the validation set; (**F**) comparison of diagnostic index of paired serum samples (pre- vs. post-surgery) of 180 lung cancer patients; (**G**) distribution of diagnostic index in the clinical subsets of the validation set. The percentages shown in the graph were sensitivities in each cancer subgroup.

Diagnostic model development was performed in the discovery set of the lung cancer dataset, which included 208 lung cancer patients and 208 non-cancer controls (Figure 1A). limma analysis was used to evaluate the statistical significance of differential miRNA expression between lung cancer patients and non-cancer controls. The top 50 differentially expressed miRNAs were listed in Table S1. Ten-fold cross validation showed that a diagnostic model with the top 4 miRNAs ranked by adjusted *p* values (hsa-miR-5100, hsa-miR-1343-3p, hsa-miR-1290, and hsa-miR-4787-3p) would result in the best AUC in the ROC curve analysis (Figure 2A). A diagnostic index calculated by the weighted sum of the 4 miRNA expression levels and normalized to the range of zero to ten showed a near-perfect AUC value of 0.999 (Figure 2B), numerically better than the AUC of 0.993 for the original 2-miRNA model from the original publication [17] (*p* = 0.16). The cut-point of six was chosen to ensure no misclassification of the non-cancer controls in the discovery set to minimize the false positives, which resulted in 98% sensitivity and 100% specificity (Figure 2C), compared to 99% for both sensitivity and specificity for the original 2-miRNA model [17].

### 3.3. Validation of the Diagnostic Model in the Lung Cancer Validation Set

The performance of the 4-miRNA model was evaluated in the lung cancer validation set (*n* = 3328), including 1358 lung cancer patients and 1970 non-cancer controls. The 4-miRNA model achieved an AUC of 0.999 (Figure 2D), significantly better than the AUC of 0.996 for the original 2-miRNA model [17] (*p* = 0.01). The new model also resulted in 99% for both sensitivity and specificity (Figure 2E), whereas the original 2-miRNA model showed 95% sensitivity and 99% specificity [17].

Furthermore, the performance of the 4-miRNA model was assessed in clinical subsets of the validation set, as defined by clinical stage, T stage, N stage, M stage, and Histology. Across all clinical subsets, the 4-miRNA model showed sensitivities of approximately 99% or above (Figure 2G, Table 2), which were superior to the sensitivities of the original 2-miRNA model (Table 2). In particular for early stage lung cancer, e.g., for both patients with stage I lung cancer and patients with T1 tumors, the 4-miRNA model demonstrated >99% sensitivity (Figure 2G, Table 2), compared to the sensitivities of 95.4 and 95.9%, respectively, for the 2-miRNA model (Table 2). In the prevalent histological types of adenocarcinoma and squamous cell carcinoma, the 4-miRNA model also demonstrated superior performance (Figure 2G, Table 2), compared to the original 2-miRNA model (Table 2).

Data on paired serum samples (pre- vs. post-surgery) were also available for 180 patients. The diagnostic indices of the 4-miRNA model for post-surgery serum samples were reduced to normal levels below the diagnostic index cut-point (Figure 2F).

### 3.4. Application of the Diagnostic Model in Additional Cancer Types

**Figure 3:**
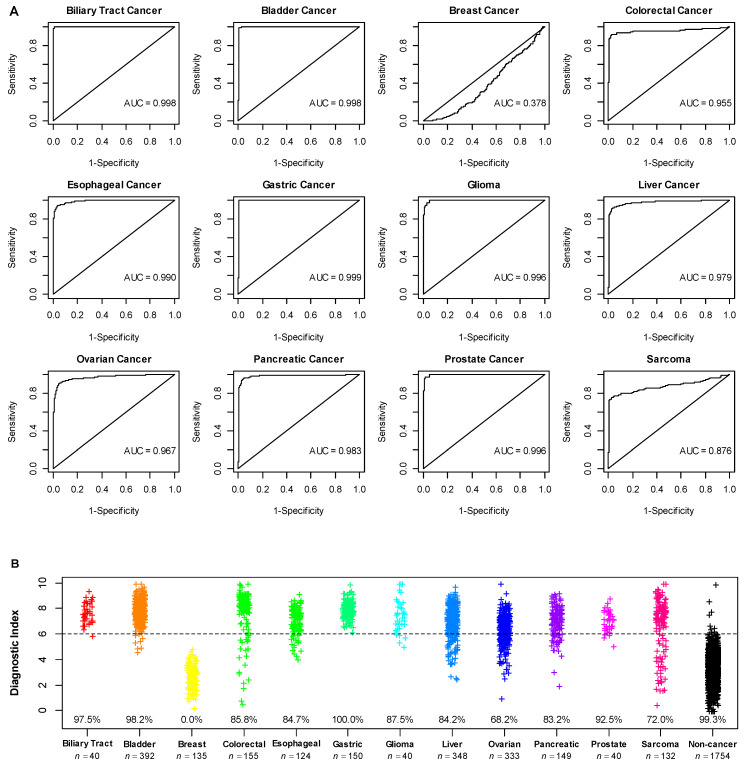
Performance of 4-miRNA diagnostic model in the datasets of additional cancers. (**A**) ROC analysis; (**B**) distribution of diagnostic index the 4-miRNA model. The percentages shown in the graph were sensitivities of each cancer type and specificity of non-cancer controls. Different colors denoted different subject conditions.

The performance of the 4-miRNA model was further assessed in the combined dataset of 3792 patients, including 2038 cancer patients across 12 different cancer types and 1754 non-cancer controls. The bladder, liver, and ovarian cancers had the largest sample sizes with >300 patients in each. Except for breast cancer in which the 4-miRNA model did not perform, the 4-miRNA model showed very strong performances with AUCs > 0.95 in biliary tract, bladder, colorectal, esophageal, gastric, glioma, liver, ovarian, pancreatic, and prostate cancers, and an AUC of 0.876 in Sarcoma (Figure 3A). Accordingly, the 4-miRNA model demonstrated high sensitivities in the range from 83.2 to 100% for biliary tract, bladder, colorectal, esophageal, gastric, glioma, liver, pancreatic, and prostate cancers, and reasonable sensitivities of 68.2 and 72.0% for ovarian cancer and sarcoma, respectively (Figure 3B). In addition, for the 1754 non-cancer controls independent of those included in the lung cancer dataset, the 4-miRNA model maintained a high specificity of 99.3%.

A further sensitivity analysis with an adjusted diagnostic index cut-point of 5.1 that would lower the specificity to 95% resulted in increased sensitivities across all 11 cancer types, demonstrating sensitivities of >90% across ten cancer types with the exception of 76.5% sensitivity for sarcoma (Table 3).

## 4. Discussion

In this study, we report on the development and performance evaluation of a 4-miRNA diagnostic model for multi-cancer early detection. We demonstrated that in the large independent set of 7120 participants including 3396 cancer patients and 3724 non-cancer individuals, the 4-miRNA model can detect 12 cancer types (biliary tract, bladder, colorectal, esophageal, gastric, glioma, live, lung, ovarian, pancreatic, prostate, and sarcoma) simultaneously with high sensitivities (80–100% for ten cancer types, and ~70% for two cancer types) while still maintaining a very high specificity of 99% that is typically required for a screening test to be useful in at-risk general population. To our knowledge, this is the first MCED diagnostic model based on circulating cell-free microRNAs. It is interesting to note that the diagnostic index for lung cancer patients decreased to the levels of non-cancer controls after tumor resection, suggesting that the diagnostic model might have the potential to be used to monitor future tumor recurrence. While experimental investigation of the biological roles of the four microRNAs in carcinogenesis is beyond the scope of the current study, two of the four miRNAs had been studied previously in cancer. hsa-miR-5100 has been found to be overexpressed in cancers of lung, gastric, pancreatic, and oral squamous cell carcinoma [22,23,24,25,26], while hsa-miR-1290 was overexpressed in cancers of colorectal, lung, glioma, and oral squamous cell carcinoma [27,28,29,30].

Noninvasive screening tests analyzing circulating nucleic acids and/or proteins have become the driving force of the MCED campaign with significant progress being made recently. Nearly all of the tests that are being developed for MCED are based on the evaluation of circulating tumor DNAs, and most utilize next generation bisulfite sequencing technology to evaluate the methylation patterns of these tumor DNAs [31,32,33,34]. Two such tests, Galleri and PanSeer, are developed as methylation-based epigenetic signatures [31,33]. In the analysis of the case-control study of the Circulating Cell-free Genome Atlas (CCGA), Galleri interrogated >100,000 methylated regions and showed that the sensitivity for 12 pre-specified cancers (anus, bladder, colon/rectum, esophagus, head and neck, liver/bile-duct, lung, lymphoma, ovary, pancreas, plasma cell neoplasm, stomach) was 67.6% for patients with stage I–III disease (*n* = 874) and increased to 76.3% (*n* = 1346) when stage IV cancer was included, while reaching a 99.3% specificity based on 1254 non-cancer controls [31]. On the other hand, PanSeer assay which targeted only 477 methylated genomic regions retrospectively analyzed plasma samples from a group of asymptomatic individuals enrolled in a longitudinal cancer monitoring study, and demonstrated a high sensitivity of 95% in 98 individuals who later were diagnosed with one of five cancers (stomach, esophageal, colorectal, lung, and liver cancer) within four years of blood draw (pre-diagnosis samples), but with a lower specificity of 96% in 207 healthy controls [33]. However, what was puzzling with PanSeer was that when it was evaluated in 113 post-diagnosis plasma samples, the test only showed a lower 88% sensitivity [33]. Another test called DELFI, based on the genome-wide analysis of cell-free DNA fragmentation patterns by next generation sequencing, achieved a 73% sensitivity across seven cancers (*n* = 208, breast, bile duct, colorectal, gastric, lung, ovarian, and pancreatic) and 98% specificity (*n* = 215) [34]. Finally, CancerSEEK, a test combining the measurement of nine protein biomarkers and detection of mutations of 16 genes in circulating cell-free DNA, showed ten-fold cross-validations and median 70% sensitivity (*n* = 1005) across eight cancers (*n* = 1005, ovary, liver, stomach, pancreas, esophagus, colorectum, lung, and breast) and 99% specificity (*n* = 812) [32]. In summary, the current MCED tests in development generally showed sensitivities in the range of 60–70% when a high specificity of 99% was mandated. Compared to these tests, our diagnostic model was much simpler with only 4 miRNAs and yet demonstrated substantially higher sensitivities in the range of 80–100% for 10 out of 12 cancer types studied with a large cohort of over 7000 participants. It is worthy of note that a simple diagnostic model not only costs significantly lower, but also can be developed into an in vitro diagnostic (IVD) test using conventional technology platform such as RT-PCR capable of decentralized testing, which has an advantage over NGS-based tests that are usually implemented as a laboratory developed test (LDT). These characteristics are important to drive the wide adoption and compliance of MCED tests as they are intended to target high-risk or at-risk general public.

Among the 13 cancer types examined in this study, only breast cancer was not detected successfully by the 4-miRNA diagnostic model. While the reason for this underperformance was not clear, it may indicate that breast cancer has a different miRNA expression profile and/or different shedding pattern of miRNAs into the bloodstream. Interestingly, Galleri and CancerSEEK also showed poor sensitivity of 30.5 and 33% in breast cancer, respectively [31,32]. Nevertheless, the poor performance in breast cancer may not be clinically important because mammography screening has been very effective in detecting early stage breast cancer and decreasing breast cancer mortality [35].

The ultimate diagnostic performance and clinical value of these MCED tests has to be established in large prospective screening trials with asymptomatic individuals. In the DETECT-A trial enrolling more than 10,000 asymptomatic women, 96 cancers were identified across ten cancer types, CancerSEEK showed a sensitivity of 27%, and that increased to 52% when adding those detected by standard-of-care screening tests [36]. In addition, CancerSEEK, when combined by PET-CT scan, showed a specificity of 99.6% and a positive predictive value (PPV) of 40.6%. On the other hand, in the interim analysis of 4033 participants from the prospective PATHFINDER study of Galleri test, 40 had a positive test result, 18 of them were confirmed to have cancer leading to a PPV of 45% [37]. For our 4-miRNA diagnostic model, assuming 1% cancer incidence rate and a conservative average sensitivity of 85 and 99.3% specificity, our model would provide a PPV of 55% when screening asymptomatic individuals. This is significantly higher than the PPVs for the four USPSTF recommended single cancer screenings, which range from 3.7 to 4.4% [38,39,40].

Our study has limitations. First, while we have access to the detailed patient-level clinical data for lung, bladder, ovarian, and liver cancer patients in the respective datasets, the clinical stage information is only available for lung, ovarian, and liver cancer patients. The sensitivity for stage I lung, ovarian, and liver cancer was 99.6, 71.6, and 87.2%, respectively, similar to the sensitivities for all lung, ovarian, and liver cancer patients. In addition, the sensitivities for biliary tract, bladder, and gastric cancers were very high (>95%), indicating the performance for these cancers is likely to be high even in early stage cancers. This is in contrast to other MCED tests where the sensitivity for early stage cancers was often substantially lower than late stage ones. For example, Galleri and CancerSEEK detected stage I cancers with a sensitivity of 16.8 and 43%, respectively [31,32]. Second, demographic data were lacking for non-cancer individuals in the combined dataset of other additional cancer types, thus we could not evaluate the potential bias due to the imbalance of such factors between cancer vs. non-cancer for those cancer types. However, such information was available for the lung cancer dataset. Analysis of ROC curves and the distribution of diagnostic index across age and gender groups in the lung cancer dataset showed no apparent bias in the performance of 4-miRNA model due to age and gender imbalance (Figure S1). Individual patient-level smoking information in the lung cancer dataset was not provided by the original publication. However, given that smoking status was matched in the discovery set to minimize potential miRNA selection bias and the near perfect performance of the 4-miRNA model in the lung cancer dataset, smoking was unlikely to significantly impact the diagnostic performance. Third, the cancer patients and the non-cancer individuals were recruited from different hospitals with different serum storage conditions. Among the 1754 non-cancer controls in the combined cancer dataset, 139 were from the same hospital (NCCH) as the cancer patients, and they did not show significantly different diagnostic index from those recruited from the other two hospitals (mean diagnostic index: 3.25 vs. 3.33 for NCCH vs. non-NCCH, *p* = 0.41). Therefore, different serum storage conditions may not contribute apparent bias in the results. Finally, our current diagnostic model is developed to detect cancers, but is not able to identify the tissue of origin. Research is currently ongoing to identify tissue-specific miRNAs that allow the prediction of tissue of origin. In addition, the 4-miRNA diagnostic model will be used together with PET scan and other imaging technology to not only confirm, but also localize the cancers, similar to how CancerSEEK was used in cancer screening [36].

We acknowledge that our study was a bioinformatics analysis of publicly available microarray datasets. While we were able to develop and evaluate the performance of a 4-miRNA diagnostic model for lung cancer and subsequently other additional cancers, wet-bench experimental investigations of the roles of the four microRNAs in carcinogenesis will provide mechanistic understanding of the cancer prediction power of these miRNAs. Furthermore, recruiting an additional cohort using a comparable or alternative technical platform to further validate the 4-miRNA model will be a critical step before the model could find its way towards clinical application. These will be the focus of our future work, which is beyond the scope of the current study.

## 5. Conclusions

In summary, our study has provided proof-of-concept data for a simple and affordable blood-based diagnostic test that detects multiple cancers. The 12 cancer types that were detected in this study account for almost 380,000 (~62%) estimated cancer deaths in the US in 2021 [2]. While the early detection of these cancers should conceivably reduce the cancer-related deaths, the ultimate determination of clinical performance and clinical utility will require the evaluation in large prospective studies with asymptomatic individuals from the intended use population.

## 6. Patents

A.Z. and H.H. have a pending patent on a microRNA-based diagnostic model for multi-cancer early detection, based on the data included in this article.

## Figures and Tables

**Table 1:** Patient and tumor characteristics for patients with lung, bladder, ovarian, and liver cancers and demographic information of the corresponding controls.

	Lung Cancer [17] *	Characteristics	Bladder Cancer [19] *	Characteristics	Ovarian Cancer [18] *	Characteristics	Liver Cancer [20] *
	*n* = 1566		*n* = 392		*n* = 333		*n* = 348
**Age (mean, SD)**	65 (10)	**Age (mean, SD)**	68 (11)	**Age (mean, SD)**	57 (12)	**Age (mean, SD)**	68 (9)
**Gender**		**Gender**		**Stage**		**Gender**	
**Male**	895 (57%)	**Male**	283 (72%)	**I**	82 (25%)	**Male**	268 (78%)
**Female**	671 (43%)	**Female**	109 (28%)	**II**	33 (10%)	**Female**	77 (22%)
**Smoking**		**Urinary cytology**		**III-IV**	218 (65%)	**unknown**	3
**Former/current**	972 (62%)	**Class I**	36 (10%)	**Histology**		**Stage**	
**Never**	594 (38%)	**Class II**	115 (31%)	**Serous**	182 (55%)	**I**	123 (37%)
**Histology**		**Class III**	73 (19%)	**Clear cell**	64 (19%)	**II**	108 (33%)
**Adenocarcinoma**	1217 (78%)	**Class IV**	50 (13%)	**Endometrioid**	43 (13%)	**III**	80 (24%)
**Squamous**	221 (14%)	**Class V**	103 (27%)	**Mucinous**	14 (4%)	**IV**	19 (6%)
**Adenosquamous**	18 (1%)	**unknown**	15	**Other epithelial**	17 (5%)	**unknown**	18
**Small cell**	23 (1%)	**T stage**		**Non-epithelial**	13 (4%)	**Child-Pugh**	
**Other**	87 (6%)	**<pT2**	300 (77%)			**A**	303 (88%)
**Stage**		**≥pT2**	90 (23%)	**Non-Cancer (*n* = 2759)**		**B**	40 (12%)
**I**	1126 (72%)	**unknown**	2	**Info Not Available**		**unknown**	5
**II**	233 (15%)	**Grade**				**Virus**	
**III-IV**	203 (13%)	**Low**	77 (20%)			**HBsAg+**	57 (16%)
**0**	4 (0%)	**High**	315 (80%)			**HCVAb+**	141 (41%)
		**Nodal status**				**non-B non-C**	147 (43%)
**Non-Cancer (*n* = 2178)**		**N+**	42 (12%)			**unknown**	3
**Age (mean, SD)**	51 (11)	**N0**	320 (88%)				
**Gender**		**unknown**	30			**Non-Cancer (*n* = 1033)**	
**Male**	1129 (52%)	**M stage**				**Age (mean, SD)**	65 (10)
**Female**	1049 (48%)	**M1**	17 (5%)			**Gender**	
**Smoking**		**M0**	347 (95%)			**Male**	239 (23%)
**Former/current**	482 (22%)	**Unknown**	28			**Female**	794 (77%)
**Never**	1696 (78%)						
		**Non-Cancer (*n* = 100)**					
		**Age (mean, SD)**	64 (16)				
		**Gender**					
		**Male**	48 (48%)				
		**Female**	52 (52%)				

* Adapted from references [17], [19], [18], [20], respectively.

**Table 2:** Comparison of sensitivities in the lung cancer clinical subsets between the original 2-miRNA model and the new 4-miRNA model.

Clinical Subsets		*n*	Original 2-miRNA Model	New 4-miRNA Model	*p*-Value *
**Clinical Stage**	**IA**	686	96.1%	99.6%	<0.001
	**IB**	285	93.7%	99.6%	<0.001
	**IIA**	146	97.3%	97.9%	0.99
	**IIB**	61	96.7%	98.4%	0.99
	**IIIA**	164	90.2%	99.4%	<0.001
	**IIIB**	6	83.3%	100.0%	0.99
	**IV**	8	100.0%	100.0%	1.00
**T Stage**	**T1a**	466	96.1%	99.6%	<0.001
	**T1b**	297	95.6%	99.3%	0.003
	**T2a**	435	93.6%	99.1%	<0.001
	**T2b**	52	92.3%	100.0%	0.134
	**T3**	89	94.4%	98.9%	0.221
	**T4**	17	94.1%	100.0%	0.99
**N Stage**	**N0**	1047	95.5%	99.5%	<0.001
	**N1**	166	95.8%	98.2%	0.289
	**N2**	142	90.1%	99.3%	<0.001
**M Stage**	**M0**	1348	94.7%	99.3%	<0.001
	**M1a**	8	100.0%	100.0%	1.00
**Histology**	**ADC**	1038	95.1%	99.2%	<0.001
	**SqCC**	205	94.2%	99.5%	0.006
	**LCC**	34	97.1%	100.0%	0.99
	**SCLC**	22	90.9%	100.0%	0.480
	**Others**	57	96.5%	100.0%	0.480

* *p* values calculated by McNemar Test.

**Table 3:** Comparison of sensitivities of the 4-miRNA diagnostic model in additional cancer datasets based on the default cut-point vs. alternative cut-point that resulted in 95% specificity.

	Default Cut-Point Based on 99% Specificity	Alternative Cut-Point Based on 95% Specificity
**Biliary Tract Cancer**	97.5%	100.0%
**Bladder Cancer**	98.2%	99.2%
**Colorectal Cancer**	85.8%	91.6%
**Esophageal Cancer**	84.7%	95.2%
**Gastric Cancer**	100.0%	100.0%
**Glioma**	87.5%	97.5%
**Liver Cancer**	84.2%	92.5%
**Ovarian Cancer**	68.2%	90.1%
**Pancreatic Cancer**	83.2%	95.3%
**Prostate Cancer**	92.5%	97.5%
**Sarcoma**	72.0%	76.5%

## Data Availability

All individual patient data were made publicly available by the original study authors. Gene Expression Omnibus (GEO) accession IDs for the datasets used in this study are included in the Methods section.

## Supplementary Materials

The following supporting information can be downloaded at: https://www.mdpi.com/article/10.3390/cancers14061450/s1, Figure S1: ROC analysis and distribution of diagnostic index across age and gender groups in the lung cancer dataset; Table S1: Top 50 differentially expressed miRNAs from the lung cancer discovery set.
