# Supplementary material for: A Novel Blood-Based microRNA Diagnostic Model with High Accuracy for Multi-Cancer Early Detection
Source: Cancers (Basel). 2022 Mar 11;14(6):1450. doi: 10.3390/cancers14061450 (PMC8946599; doi:10.3390/cancers14061450)
Supplement: Supplementary file 1 [file cancers-14-01450-s001.zip › cancers-1570865-supplementary.pdf]

cancers MDPI

Supplementary Files

A Novel Blood-Based microRNA Diagnostic Model with High Accuracy for Multi-Cancer Early Detection

Andrew Zhang and Hai Hu

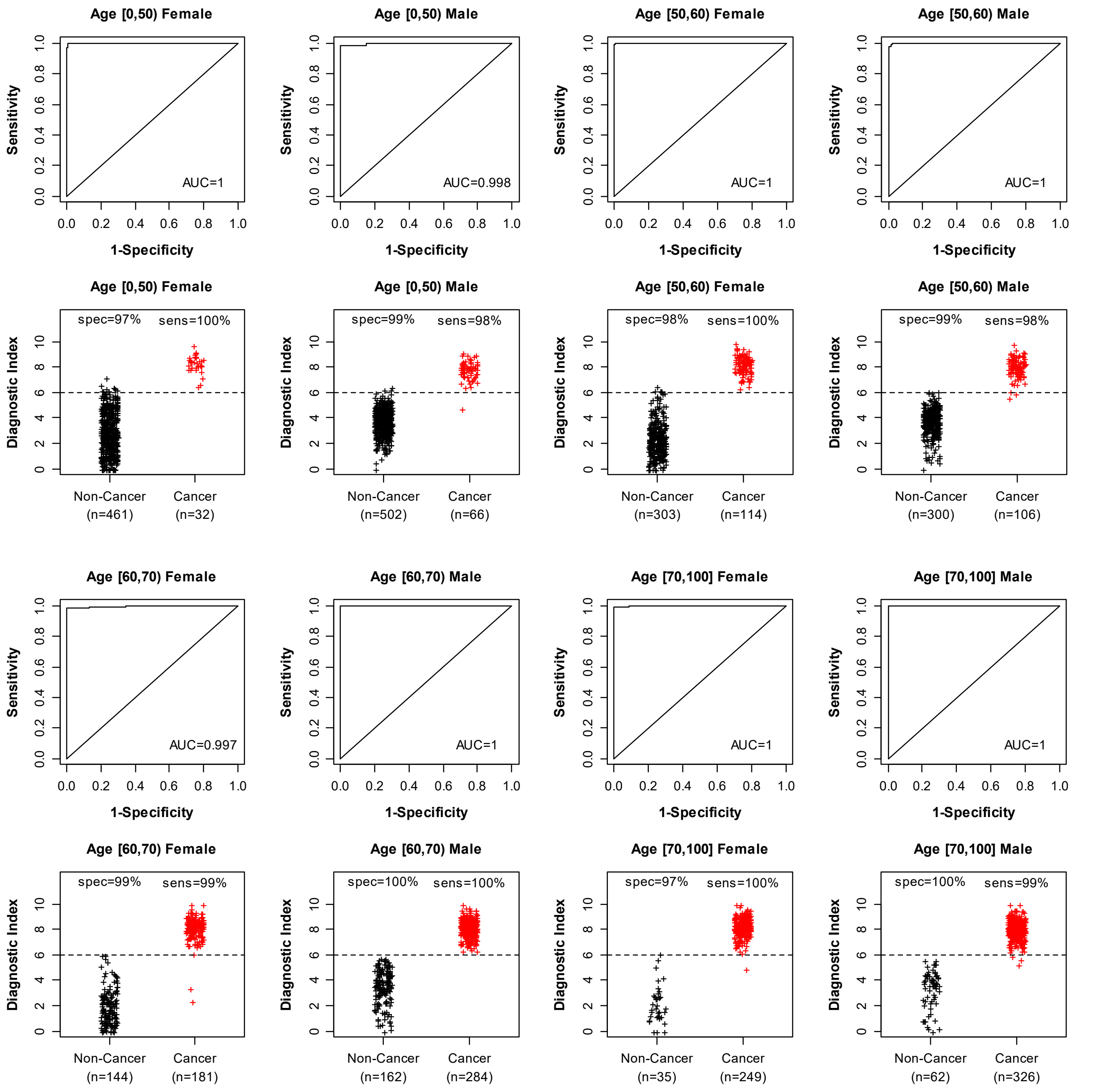

Figure S1. ROC analysis and distribution of diagnostic index across age and gender groups in the lung cancer dataset.

Table S1. Top 50 differentially expressed miRNAs from the lung cancer discovery set.

miRBase Accession ID	Log Fold Change	Adjusted *p* Value	AUC of ROC	miRNA Names
MIMAT0022259	3.93	9.99×10^{-176}	1.00	hsa-miR-5100
MIMAT0019776	2.61	5.83×10^{-94}	0.97	hsa-miR-1343-3p
MIMAT0005880	6.54	2.22×10^{-87}	1.00	hsa-miR-1290
MIMAT0019957	1.85	6.81×10^{-67}	0.94	hsa-miR-4787-3p
MIMAT0027654	1.36	1.37×10^{-63}	0.95	hsa-miR-6877-5p
MIMAT0000071	4.09	1.66×10^{-62}	0.93	hsa-miR-17-3p
MIMAT0027430	-0.69	5.09×10^{-62}	0.93	hsa-miR-6765-5p
MIMAT0018925	-0.62	1.45×10^{-61}	0.94	hsa-miR-1268b
MIMAT0016879	1.78	6.45×10^{-59}	0.90	hsa-miR-4258
MIMAT0001631	5.01	3.71×10^{-58}	0.94	hsa-miR-451a
MIMAT0005582	-0.78	1.01×10^{-57}	0.92	hsa-miR-1228-5p
MIMAT0031000	2.09	6.42×10^{-55}	0.91	hsa-miR-8073
MIMAT0018976	1.66	3.91×10^{-51}	0.90	hsa-miR-4454
MIMAT0004561	1.04	1.90×10^{-50}	0.91	hsa-miR-187-5p
MIMAT0016916	1.59	1.05×10^{-49}	0.88	hsa-miR-4286
MIMAT0027392	1.35	1.53×10^{-49}	0.87	hsa-miR-6746-5p
MIMAT0005867	1.20	9.31×10^{-49}	0.89	hsa-miR-663b
MIMAT0023700	0.79	2.77×10^{-47}	0.89	hsa-miR-6075
MIMAT0021021	0.80	3.16×10^{-46}	0.88	hsa-miR-5001-5p
MIMAT0027478	0.68	6.98×10^{-46}	0.89	hsa-miR-6789-5p
MIMAT0019050	1.06	1.19×10^{-45}	0.89	hsa-miR-4513
MIMAT0015076	4.11	1.76×10^{-45}	0.86	hsa-miR-3192-5p
MIMAT0030987	3.50	1.77×10^{-45}	0.88	hsa-miR-8060
MIMAT0026636	2.75	2.02×10^{-45}	0.89	hsa-miR-668-5p
MIMAT0005922	-0.61	2.40×10^{-45}	0.87	hsa-miR-1268a
MIMAT0022742	1.45	2.67×10^{-45}	0.86	hsa-miR-1273g-3p
MIMAT0019806	1.06	5.43×10^{-45}	0.85	hsa-miR-4706
MIMAT0000422	3.73	5.43×10^{-45}	0.89	hsa-miR-124-3p
MIMAT0015041	1.28	9.38×10^{-45}	0.86	hsa-miR-1260b
MIMAT0019869	3.16	9.50×10^{-45}	0.89	hsa-miR-4740-5p
MIMAT0005792	2.32	1.08×10^{-44}	0.89	hsa-miR-320b
MIMAT0031180	1.27	4.78×10^{-43}	0.87	hsa-miR-7977
MIMAT0000100	4.10	1.07×10^{-42}	0.86	hsa-miR-29b-3p
MIMAT0019810	2.78	2.73×10^{-42}	0.86	hsa-miR-4708-3p
MIMAT0019064	2.39	3.12×10^{-42}	0.85	hsa-miR-4525
MIMAT0003218	2.49	3.43×10^{-42}	0.87	hsa-miR-92b-3p
MIMAT0016878	1.01	4.69×10^{-42}	0.86	hsa-miR-4257
MIMAT0019848	2.68	7.55×10^{-42}	0.86	hsa-miR-4727-3p
MIMAT0000092	2.01	9.49×10^{-42}	0.86	hsa-miR-92a-3p
MIMAT0003326	1.08	1.02×10^{-41}	0.84	hsa-miR-663a
MIMAT0027474	1.23	5.33×10^{-41}	0.83	hsa-miR-6787-5p
MIMAT0014996	1.19	7.21×10^{-41}	0.85	hsa-miR-3131
MIMAT0027504	0.85	2.03×10^{-40}	0.84	hsa-miR-6802-5p
MIMAT0003330	2.54	3.90×10^{-40}	0.87	hsa-miR-654-5p
MIMAT0025847	1.93	1.70×10^{-39}	0.89	hsa-miR-6511b-5p
MIMAT0004514	4.29	1.38×10^{-38}	0.83	hsa-miR-29b-1-5p
MIMAT0018929	0.42	1.66×10^{-38}	0.88	hsa-miR-4417

MIMAT0019862	1.51	2.07×10^{-38}	0.87	hsa-miR-4736
MIMAT0027583	0.91	3.82×10^{-38}	0.84	hsa-miR-6840-3p
MIMAT0019815	2.58	4.97×10^{-38}	0.85	hsa-miR-4710
